# Mesenchymal Stem Cells Support Survival and Proliferation of Primary Human Acute Myeloid Leukemia Cells through Heterogeneous Molecular Mechanisms

**DOI:** 10.3389/fimmu.2017.00106

**Published:** 2017-02-09

**Authors:** Annette K. Brenner, Ina Nepstad, Øystein Bruserud

**Affiliations:** ^1^Department of Clinical Science, Section for Hematology, University of Bergen, Bergen, Norway; ^2^Department of Medicine, Haukeland University Hospital, Bergen, Norway

**Keywords:** acute myeloid leukemia, mesenchymal stem cells, proliferation, apoptosis, cytokines, chemokines

## Abstract

Acute myeloid leukemia (AML) is a bone marrow malignancy, and various bone marrow stromal cells seem to support leukemogenesis, including osteoblasts and endothelial cells. We have investigated how normal bone marrow mesenchymal stem cells (MSCs) support the *in vitro* proliferation of primary human AML cells. Both MSCs and primary AML cells show constitutive release of several soluble mediators, and the mediator repertoires of the two cell types are partly overlapping. The two cell populations were cocultured on transwell plates, and MSC effects on AML cells mediated through the local cytokine/soluble mediator network could thus be evaluated. The presence of normal MSCs had an antiapoptotic and growth-enhancing effect on primary human AML cells when investigating a group of 51 unselected AML patients; this was associated with increased phosphorylation of mTOR and its downstream targets, and the effect was independent of cytogenetic or molecular-genetic abnormalities. The MSCs also supported the long-term proliferation of the AML cells. A subset of the patients also showed an altered cytokine network with supra-additive levels for several cytokines. The presence of cytokine-neutralizing antibodies or receptor inhibitors demonstrated that AML cells derived from different patients were heterogeneous with regard to effects of various cytokines on AML cell proliferation or regulation of apoptosis. We conclude that even though the effects of single cytokines derived from bone marrow MSCs on human AML cells differ among patients, the final cytokine-mediated effects of the MSCs during coculture is growth enhancement and inhibition of apoptosis.

## Introduction

Acute myeloid leukemia (AML) is an aggressive malignancy that mainly affects the elderly; the disease is characterized by bone marrow infiltration of immature leukemic blasts (1), and it is highly heterogeneous with respect to leukemia cell biology as well as response to therapy (2). Approximately 50–60% of AML patients carry clonal chromosomal abnormalities that reflect the chemosensitivity of the disease (3). The disease is also associated with specific gene mutations exhibiting prognostic impact, where *FMS-related tyrosine kinase 3* internal tandem repeats (*Flt3*-ITD; adverse prognosis) and mutations in *nucleophosmin* (*NPM1*; favorable prognosis) are the most prominent (4).

Mesenchymal stem or stromal cells (MSCs) are capable of self-renewal and differentiation into osteoblasts, chondrocytes, or adipocytes (5), the most immature MSCs can also trans-differentiate into other embryonic lineages (6, 7). The cells can be isolated from almost any kind of connective tissue (8, 9), and bone marrow MSCs provide a microenvironment for growth, differentiation, and survival of both normal (10) and leukemic (11) hematopoietic cells. The bone marrow MSC population seems to be important in leukemogenesis (12) and also to contribute to chemoresistance through its release of specific soluble mediators (13, 14).

In this study, we have therefore characterized the cytokine-mediated crosstalk between AML cells and normal bone marrow MSCs. Due to the heterogeneity of the disease, we have investigated a large group of unselected patients. Our studies suggest that MSC-derived cytokines have antiapoptotic effects and support AML cell proliferation for most patients, but the molecular mechanisms causing these effects differ among patients.

## Materials and Methods

### AML Patient Population and Leukemic Cell Preparation

The study was approved by the local ethics committee (Regional Ethics Committee III, University of Bergen) and samples collected after written informed consent. AML blasts from peripheral blood were derived from 51 consecutive/unselected patients admitted to our department for AML therapy (22 females, 29 males; median age 67 years; range 19–87 years). A majority of 36 patients had *de novo* AML (Table 1), 4 patients had relapsed disease, and 11 patients had secondary AML.

**Table 1:** **Biological and clinical characteristics of the 51 acute myeloid leukemia (AML) patients included in the study**.

Patient characteristics		Cell morphology		Cell genetics	
Age		FAB classification^a^		Cytogenetics^b^	
Median (years)	67	M0	3	Favorable	9
Range (years)	19–87	M1	15	Intermediate	6
		M2	7	Normal	23
Gender		M3	3	Adverse	10
Females	22	M4	8	n.d.	3
Males	29	M5	10		
		n.d.	5	*Flt3* mutations	
Secondary AML				ITD	15
MDS	6	CD34 receptor		Wild type	26
CMML	4	Negative (≤20%)	14	n.d.	10
Chemotherapy	1	Positive (>20%)	32		
		n.d.	5	*NPM1* mutations	
AML relapse	4			Insertion	16
				Wild type	26
				n.d.	9

*^a^No patient was diagnosed with FAB M6 or M7*.

*^b^Patients with favorable karyotype had inv(16); t(15;17); and t(8;21). Patients with adverse karyotype had −5; −7; +8 and complex karyotype with at least three abnormalities*.

*n.d., not determined*.

Acute myeloid leukemia cells were isolated from peripheral blood of patients with levels of circulating blasts by density gradient separation (Lymphoprep; Axis-Shield, Oslo, Norway; specific density 1.077 g/mL). The cells were stored in liquid nitrogen until use (15).

### Reagents

The following neutralizing antibodies and receptor antagonists (all from R&D Systems, Abingdon, UK) were used at the following concentrations: (i) 100 ng/mL of affinity purified polyclonal antibodies (goat IgG) against the vascular endothelial growth factor (VEGF), hepatocyte growth factor (HGF), basic fibroblastic growth factor (bFGF), and IL-6. At this concentration, the antibodies will block ≥50% of receptor binding to VEGF (80 ng/mL), HGF (8 ng/mL), bFGF (0.1 ng/mL), and IL-6 (5 ng/mL), thus concentrations higher than the levels in our MSC cultures (16); (ii) 3 µM of the CCR1 antagonists BX471 (17) and BX513 (18); (iii) 1.5 µM of the combined CCR1 and CCR3 antagonist UCB35625 and its stereoisomer J113863 (19, 20); and (iv) 300 µM of the CXCR4 antagonist AMD3100 (13). Normal goat IgG was used in the antibody control cultures. Cytarabine (Sigma-Aldrich, St. Louis, MO, USA) was tested in dose-response experiments using concentrations between 12.5 nM and 2 µM (21).

### *In Vitro* Expansion of MSCs

Human MSCs from three healthy donors (MSC24429, MSC24539, and MSC25200) were purchased from Lonza (Cambrex BioScience, Walkersville, MD, USA). According to the distributor’s information, the cells were obtained in passage two and showed the ability to differentiate into the mesenchymal lineages. All cells tested negative for mycoplasma, bacteria, and fungi. The MSCs were expanded in complete mesenchymal stem cell growth medium (MSCGM™; Lonza), which contains 10% fetal bovine serum (FBS) and 4 mM l-glutamine; cells were trypsinized and used for the experiments in passages three or four. Our previous studies of global gene expression profiles of *in vitro* expanded MSCs showed no evidence for differentiation of such expanded MSCs (16).

### Analysis of AML Cell Proliferation and Viability in Transwell Cocultures with MSCs

#### Preparation of MSC-AML Cell Cocultures

Cultures were prepared in transwell plates (Costar 3401 plates; Costar, Cambridge, MA, USA). The MSCs (2 × 10^4^ cells/well) were seeded in the lower chamber in complete MSCGM™ medium (1 mL/well). After 3 days of culture (37°C, humidified atmosphere, 5% CO_2_) the medium was exchanged and subsequently 1 × 10^6^ AML cells were added in 0.5 mL medium to the upper chamber separated from the MSCs by a semipermeable membrane (0.4 µm pore size). The cells were cultured for 3 days, in which the MSCs did not reach confluence.

#### Analysis of Cell Proliferation by ^3^H-Thymidine Incorporation

After 2 days of coculture, 275 kBq of ^3^H-thymidine (PerkinElmer, Waltham, MA, USA) was added to the upper wells and the cells were incubated for another day. The nuclear ^3^H-thymidine incorporation was then measured by liquid scintillation counting as described in detail previously (16). All cultures were prepared in triplicates and the median counts per minute (cpm) were used for all calculations. A ^3^H-thymidine incorporation corresponding to an activity of at least 1,000 cpm was defined as detectable proliferation (22).

#### Analysis of AML Cell Viability

Acute myeloid leukemia cells and MSCs were cocultured in transwell plates for 3 days before the percentage of viable leukemic cells was determined by flow cytometry after staining with propidium iodide (PI) and fluorescein isothiocyanate-conjugated Annexin V antibodies (Tau Technologies BV, Kattendijke, the Netherlands) as described in detail previously (23). Briefly, after staining with PI/anti-Annexin V, the flow cytometric analysis could identify the viable Annexin^−^PI^−^, early apoptotic Annexin V^+^PI^−^, and late apoptotic/necrotic Annexin V^+^ PI^+^ AML cell subsets.

We also cultured primary AML cells from 10 patients in direct contact with MSCs in 6-well tissue culture plates; 20,000 MSCs were precultured for 3 days before 1 × 10^6^ primary AML cells were added to each well. AML cell viability was analyzed 20 h later before the MSCs reached confluence.

### Analysis of the Cytokine Profile in MSC-AML Cell Transwell Cocultures

Supernatants were harvested from the lower chambers and stored at −80°C. The following cytokine levels were determined by Luminex analyses or enzyme-linked immunosorbent assay (ELISA) (R&D Systems): (i) the chemokines CCL2-5 and CXCL1/5/8/10/11, (ii) the interleukins IL-1β/1RA/6/10/33, (iii) the matrix metalloproteinases MMP-1 and -2, (iv) tumor necrosis factor-α (TNFα), and (v) granulocyte colony-stimulating factor (G-CSF), granulocyte macrophage colony-stimulating factor (GM-CSF), HGF, bFGF, VEGF, and soluble angiopoietin 1 (Ang-1) receptor Tie-2.

### Long-term *In Vitro* AML-MSC Cocultures and Analysis of Colony-Forming Cells

Cocultures of MSCs with primary AML cells were prepared as described above. The blasts were transferred weekly to new upper chambers of transwell cocultures where fresh MSCs had been seeded in the lower chambers 3 days in advance. The medium was exchanged twice a week throughout the 3 weeks of culture period in transwell cocultures. After 3 weeks, the AML cells were harvested and subsequently seeded in the colony-formation assay to estimate the number of colony-forming units (CFU). We used two different colony-formation assays; MethoCult™ H4434 Classic and H4534 Classic without erythropoietin medium (StemCell Technologies, Vancouver, BC, Canada). The cells were seeded in duplicate in 24-well plates with 0.5 mL medium/well. Colonies containing more than 30 cells were scored using an inverted microscope after 14 days of *in vitro* culture.

### Analysis of Intracellular Signaling and H2AX Phosphorylation

#### Antibodies

The following antibodies were used for analysis of protein phosphorylation: Alexa Fluor^®^ 647 Mouse anti-PDPK1 (pS241), Alexa Fluor^®^ 647 Mouse anti-PKCα (pT497), Alexa Fluor^®^ 647 Mouse anti-Akt (pS473), phycoerythrin (PE) Mouse Anti-Akt (pT308), PE Mouse Anti-mTOR (pS2448), Alexa Fluor^®^ 647 Mouse anti-4E-BP1 (pT36/pT45), Alexa Fluor^®^ 647 Mouse anti-elF4E (pS209), Alexa Fluor^®^ 647 Mouse anti-S6 (pS244), PE Mouse anti-S6 (pS240), V450 Mouse anti-S6 (pS235/pS236) Alexa Fluor^®^ 647 Mouse anti-PKCα (pT497) (all from BD Biosciences, Franklin Lakes, NJ, USA), and mTOR (7C10) Rabbit mAb (Alexa Fluor^®^ 647 Conjugate) (Cell Signaling Technology, Danvers, MA, USA).

#### Preparation of Conditioned Medium

MSC24539 was cultured as described above and the conditioned medium harvested before the cells reached confluence.

#### Analysis of Protein Phosphorylation

Thawed, cryopreserved leukemic cells were incubated for 20 min in culture medium, thereafter incubated with conditioned medium (final concentration 50%) for 30 min before being directly fixed in 1.5% paraformaldehyde and thereafter permeabilized with methanol. The cells were then rehydrated by adding 2 mL phosphate-buffered saline (PBS), gently resuspended, and then centrifuged. The cell pellet was washed twice with 2 mL PBS and resuspended in 150 µL PBS + 0.1% BSA (Sigma-Aldrich). The washed cells were blocked with immunoglobulin (Octagam; Octapharma, Jessheim, Norway) and 1% BSA, and split evenly into separate tubes (1 × 10^5^ cells/sample) before staining. All staining panels included the same live dead discriminator FITC Mouse anti-Cleaved PARP (Asp214; BD Biosciences) and Alexa Fluor^®^ 647 Mouse anti-Cleaved PARP (Asp 214; BD Biosciences), as well as one blank sample. Flow cytometric analysis of protein phosphorylation was performed as described in detail previously (24).

#### Analysis of H2AX Phosphorylation

The percentage of phosphorylated compared to total H2AX was detected using a cell-based ELISA kit [Phospho-Histone H2AX (S139) Immunoassay; R&D Systems]. The assay was performed according to the manufacturer’s instruction for suspension cells; 5 × 10^4^ AML cells were incubated for 16 h in both MSC medium and MSC-conditioned medium (final concentration of 50%).

### Statistical and Bioinformatical Analyses

The statistical analyses were performed with the IBM Statistical Package for the Social Sciences (SPSS) version 23 (Chicago, IL, USA). The Wilcoxon signed-rank test was used to compare paired samples, whereas Kruskal–Wallis and Mann–Whitney *U*-tests were used to compare different groups. The χ^2^ test was used to analyze categorized data and the Kendall’s tau-b test for correlation analyses. *P*-values <0.05 were regarded as statistically significant. Supervised hierarchical clustering was performed using the J-Express 2012 software (MolMine AS, Bergen, Norway). The data were log(10) transformed prior to clustering.

## Results

### Human MSCs Increase the Proliferation of Primary Human AML Cells during *In Vitro* Coculture

**Figure 1:**
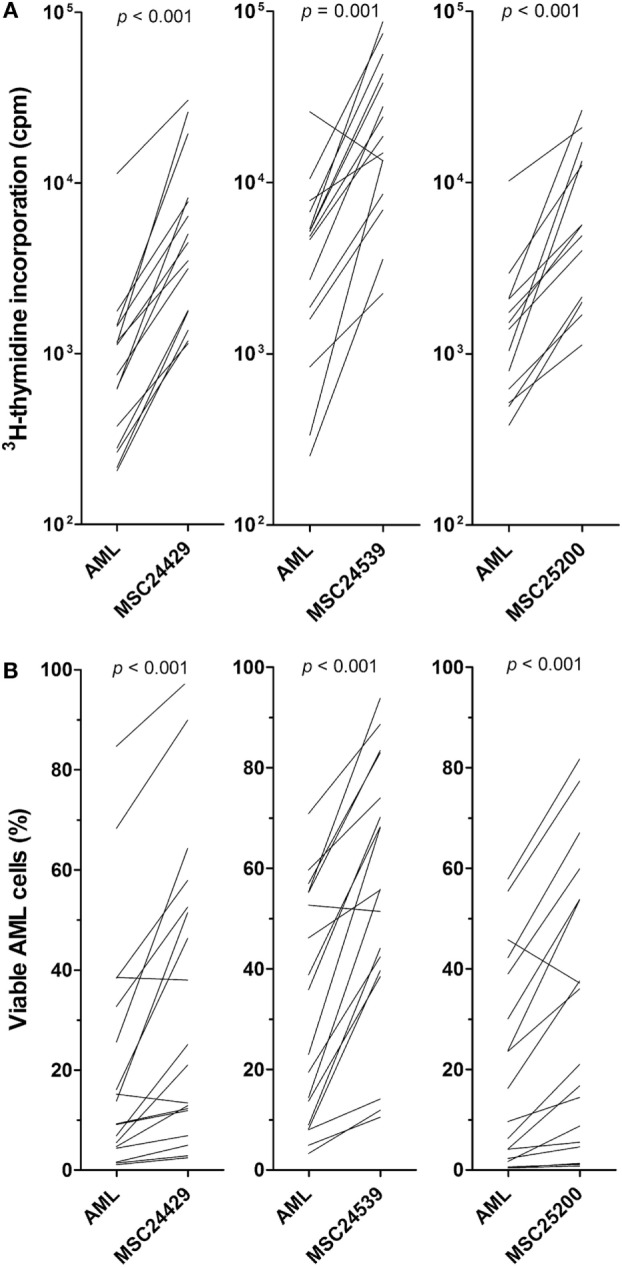
**The effect of normal bone marrow mesenchymal stem cells (MSCs) on *in vitro* proliferation and viability of primary human acute myeloid leukemia (AML) cells; a comparison of MSCs derived from three healthy donors (MSC24429, MSC24539, MSC25200)**. Primary human AML cells derived from 18 consecutive/unselected leukemia patients were cocultured with normal MSCs from the three donors. The MSCs were initially cultured for 3 days to allow them to establish adherent *in vitro* proliferation; primary human AML cells were then added and both leukemia cell proliferation and viability were assayed after additional 3 days of culture. [**(A)**, upper part] Proliferation was assayed as ^3^H-thymidine incorporation. The results for each patient are presented as nuclear thymidine incorporation (counts per minute, cpm). The *p*-value for the statistical comparison of the overall results is given at the top of the figure for each of the individual MSC. Each line represents the results for one patient. [**(B)**, lower part] Leukemia cell viability was assayed by the Annexin V-PI flow cytometric assay. The *p*-value for the statistical comparison of the overall results for each of the MSCs is given at the top of the figure for the MSCs. Each line represents the results for one patient.

Primary AML cells from 18 consecutive patients were cocultured with normal MSCs from the three different donors (MSC24429, MSC24539, and MSC25200) in transwell plates. AML cell proliferation was determined after 3 days of culture. AML cell proliferation was significantly increased by coculture with MSCs compared with the corresponding control cultures with AML cells alone (Figure 1A); this increase reached statistical significance for all three MSC donors (*p* ≤ 0.001, Wilcoxon’s signed-rank test), even though the AML cells showed undetectable proliferation for two patients, both when cultured in medium alone and in the presence of all three MSC donor cells. The other 16 patients showed increased proliferation for at least one of the three MSCs. The median increase in ^3^H-thymidine incorporation corresponded to approximately fivefold increase for each of the three donors.

### Human MSCs Have Antiapoptotic Effect on *In Vitro* Cultured Primary AML Cells

Primary AML cells show spontaneous or stress-induced apoptosis during *in vitro* culture (25). We prepared *in vitro* transwell cocultures for MSCs and AML cells derived from the same 18 patients as tested in the proliferation assay (see above). MSCs were pre-cultured for 3 days before AML cells were added and leukemic cell viability assayed after 3 days of coculture by flow cytometric analysis. The effect of all three human bone marrow MSCs was investigated (MSC24429, MSC24539, and MSC25200). The results are summarized in Table S1 in Supplementary Material and presented in detail in Figure 1B. AML cell viability showed a wide variation after three days of *in vitro* coculture (variation range 0.4–84.7% viability). When comparing the overall results, the fraction of viable cells was significantly increased (*p* < 0.001, Wilcoxon’s signed-rank test) after coculture in the presence of all three MSCs compared with the corresponding MSC-free controls, and the median fraction of viable cells was approximately doubled for each of the three MSC donors.

### The Growth-Enhancing and Antiapoptotic Effects of Human MSCs on Primary Human AML Cells—A Study of Patient Heterogeneity for a Group of 51 Consecutive/Unselected Patients

**Figure 2:**
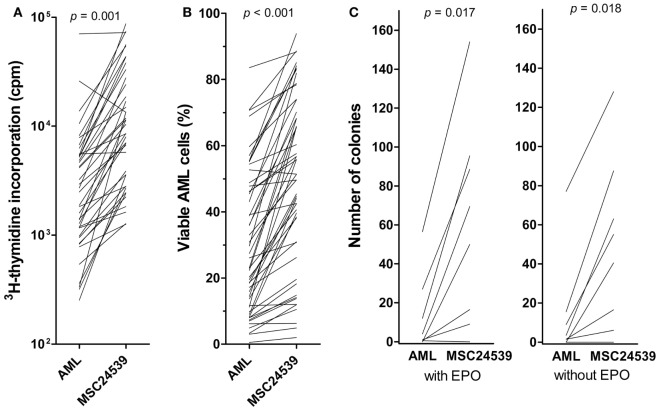
**The effects of mesenchymal stem cells (MSCs) on acute myeloid leukemia (AML) cell proliferation/viability in suspension cultures and AML cells tested in a clonogenic assay**. The effect of MSCs on the *in vitro* proliferation [**(A)**, left] and viability [**(B)**, middle] of primary human AML cells derived from 51 consecutive patients was examined. The AML cells were then cocultured with normal MSC24539. The MSCs were initially cultured for 3 days to allow them to establish adherent *in vitro* proliferation; primary human AML cells were thereafter added and **(A)** leukemia cell proliferation was assayed as ^3^H-thymidine incorporation after additional 3 days of culture; additionally, **(B)** AML cell viability was assayed by the Annexin V-PI flow cytometric assay. The *p*-values for the statistical comparison of the overall results are given at the top of the figure. Each line represents the results for one patient. [**(C)**, right] The effects of MSCs on clonogenic AML cells were also investigated. The leukemic cells were cultured either in medium alone or in transwell cocultures together with MSC24593 for 3 weeks; the number of clonogenic cells were thereafter compared for AML cells precultured in medium alone and together with MSCs. The total culture period was thus 5 weeks. The MSCs significantly increased the number of colony-forming units (*p*-values are given on top of the figure) in growth media both with and without erythropoietin. The results are presented as mean of duplicate samples (average deviation from mean corresponding to 1.3 colonies and 8.7% of the total colony number). Each line represents the results for one patient.

Because the proliferation and viability results were consistent among MSC donors, the comparison of MSC effects for a larger group of 51 consecutive patients was performed only for MSC24539. MSC-induced enhancement of AML cell proliferation in transwell cocultures was highly significant also when analyzing the overall results for this larger group of patients (Figure 2A; *p* < 0.00001, Wilcoxon’s signed-rank test). The growth enhancement showed no significant association with AML cell differentiation (morphology according to FAB classification, CD34 expression), karyotype (favorable/intermediate/adverse/normal), or *Flt3/NPM1* mutations (data not shown). Finally, cells from 11 patients showed detectable proliferation neither in medium alone nor in cocultures with MSC24539. For five additional patients, the presence of MSCs showed no or minimal growth enhancement, corresponding to less than 20% alteration and an absolute change of less than 2,000 cpm in the presence of MSCs. These 16 patients differed significantly from the other 35 patients with regard to cell differentiation as CD34^−^ cells showed weaker proliferation in the presence of MSCs than CD34^+^ cells (8/14 CD34^−^ patients with non-proliferating cells in contrast to only 6/32 CD34^−^ patients with proliferating cells; *p* = 0.011, χ^2^ likelihood ratio) (data not shown).

The same 51 patients were also tested in transwell cocultures with regard to a MSC-associated antiapoptotic effect; viability analyzed by the Annexin V-PI assay was then compared for AML cells cultured in medium alone or cocultured with MSC24539 for 3 days. The presence of MSCs increased AML cell viability significantly also when testing this extended patient group (Figure 2B; *p* < 0.00001, Wilcoxon’s signed-rank tests). This antiapoptotic effect showed no significant association with AML cell differentiation, karyotype, or *Flt3/NPM1* mutations (data not shown). However, proliferation <1,000 cpm in the presence of MSCs seemed to be associated with weaker antiapoptotic effects of MSCs; i.e., 8/21 patients with viability increase <10% points and 0/20 patients with an increase >20% points showed undetectable proliferation in cocultures (*p* = 0.001, χ^2^ likelihood ratio).

Because primary AML cells derived from 16 patients showed undetectable proliferation during coculture with MSCs, we also analyzed separately the effect of MSCs on AML cell viability for these patients. However, when analyzing the overall results, a highly significant increase in AML cells viability (*p* < 0.001) after MSC coculture was seen also for these 16 patients with non-proliferating AML cells, and a >10% point increase was seen for eight of these patients (see above). Thus, the MSC effect on AML cell viability is not only caused by the increased proliferation but also by additional effects possibly affecting the balance between pro- and antiapoptotic signaling.

### Normal Human MSCs Support the Long-term Proliferation of Primary Human AML Cells

To further investigate the MSC effects on AML cell proliferation, we used an *in vitro* model based on 21 days of coculture; this is the same culture period as used previously by Griessinger et al. (26) in their studies of leukemia-initiating AML cells. After this period of transwell cocultures, the number of colony-forming cells was compared for leukemia cells cultured alone and cells cocultured with normal MSCs. We investigated AML cells from eight patients showing both enhanced proliferation and viability in the short-term assays described above. All except one patient showed an increased number of viable cells after 21 days of coculture with the normal MSC24539 cells (data not shown). After the initial 21 days culture period, the cells were seeded in the CFU assays. Even though the culture medium was different in the CFU assays compared with the transwell cocultures containing MSC medium, colony formation could be detected for seven of the eight patients. For all these seven patients, we observed an increased number of CFUs in the AML cell populations previously cocultured with MSCs compared with the corresponding control cultures where AML cells were cultured alone without MSCs in the lower transwell chamber (Figure 2C). Thus, the MSC-associated growth enhancement also includes long-term proliferating AML cells.

### Normal Human MSCs Have Antipoptotic Effects on Primary Human AML Cells Also in the Presence of Cytarabine

We investigated the effect of 50 nM cytarabine on primary human AML cells derived from 10 patients. All patients showed an increase in leukemia cell viability corresponding to >20% in presence of MSC24539. The cytarabine concentration was chosen based on dose-response (2 µM, 0.5 µM, 125 nM, 50 nM, and 12.5 nM) pilot experiments, which showed that cytarabine at 50 nM decreased AML cell viability for a marked subset of patients when using our *in vitro* models (Figure S1 in Supplementary Material). These results show that there is heterogeneity among patients with regard to the proapoptotic effect of cytarabine, and decreased AML cell viability was seen only for a subset of patients when testing cytarabine at concentrations 12.5–500 nM and with a large overlap between the effects of 50 and 500 nM. At the same time, the drug had a clear antiproliferative effect even at 50 nM and at higher concentrations no detectable cytokine-dependent proliferation could be detected (data not shown). Finally, the concentrations 50–500 nM correspond to levels reached *in vivo* and 50 nM is close to the steady-state concentrations seen during conventional AML induction treatment and higher than the levels reached during low-dose subcutaneous cytarabine treatment (27); both these therapeutic strategies for cytarabine treatment can induce complete remissions (27, 28).

**Figure 3:**
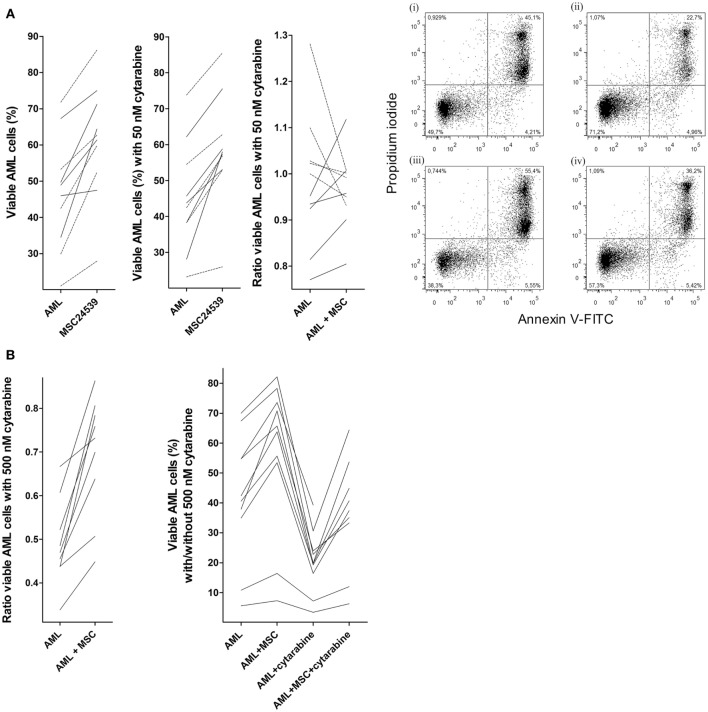
**Effects of mesenchymal stem cells (MSCs) on primary human acute myeloid leukemia (AML) cells treated with cytarabine; (A) effect of 50 nM cytarabine-exposure on AML cell viability in transwell cocultures and (B) effect of 500 nM cytarabine tested in direct-contact cocultures**. Primary AML cells derived from the same 10 patients were included in all these studies, and MSC24539 was used in all experiments. The AML cell viability was analyzed by flow cytometry. [**(A)**, transwell cocultures] For each patient, we compared cultures containing AML cells alone (AML) or AML cells plus MSCs (AML + MSC). The three figures from left to the middle right show (i) AML cell viability for cells cultured in medium alone with or without MSCs; (ii) AML cell viability in the presence of 50 nM cytarabine for leukemic cells cultured with and without MSCs; and (iii) a comparison of the medium culture ratio (i.e., viable cells in cytarabine-containing cultures versus drug-free controls; medium alone) and MSC culture ratio (i.e., viable cells in cytarabine-containing cultures versus drug-free controls; MSCs being added to both cultures). The results for the five patients (solid lines) for which 50 nM cytarabine exhibited a proapoptotic effect in the presence of MSCs are indicated. The results for one responding patient are presented in detail (right part of the figure; i/ii: drug-free control, iii/iv: 50 nM cytarabine without and with MSCs, respectively); the percentage of viable cells (population at the lower left) is indicated in the figures for each of the four cultures. [**(B)**, direct-contact cultures] The left part of the figure compares the medium culture ratio (i.e., viable cells in cytarabine-containing cultures versus drug-free controls; medium alone) and MSC culture ratio (i.e., viable cells in cytarabine-containing cultures versus drug-free controls; MSCs being added to both cultures) when testing 500 nM cytarabine. The right part of the figure shows the percentage of viable primary AML cells (10 patients tested, one patient not tested for AML cells + cytarabine + MSC) cultured in medium alone, together with MSCs, in medium containing 500 nM cytarabine, and together with 500 nM cytarabine and MSCs.

Primary AML cells were cultured in transwell cultures for 20 h either alone or in coculture with MSC24539, and cultures with/without MSCs were prepared with and without 50 nM cytarabine (Figure 3A). The present independent experiments confirmed that patients are heterogeneous with regard to susceptibility to cytarabine; a reduction in the number of viable cells exceeding 5% was seen for 5 out of 10 patients in the present experiments and for 16 out of 32 patients in the previous dose-response studies. Furthermore, the percent cytarabine-induced decrease of AML cell viability for the five patients in the present study was significantly higher for cells cultured in medium alone (median decrease 9%, range 6–23%) compared with cells in coculture with MSC24539 (median decrease 4%, range 12–20%; *p* = 0.045). Finally, increased AML cell viability was seen for all 10 patients in MSC cocultures, both in presence and absence of 50 nM cytarabine.

We then compared the overall effects of MSCs on AML cell viability, i.e., leukemic cells cultured in direct contact with MSCs, on the viability of primary human AML cells derived from the same 10 patients as used above. Cytarabine was tested at 50 and 500 nM, the last concentration corresponding to the highest steady-state levels seen during conventional doses of 100–200 mg/m^2^ as daily continuous intravenous infusions (27). The presence of MSCs caused a comparable increase in AML cell viability in these direct-contact experiments as described above for the transwell cocultures and similar to the transwell cocultures 50 nM cytarabine induced reduction of cell viability for five patients also in these direct-contact experiments (data not shown). In contrast, 500 nM cytarabine caused a significant reduction in AML cell viability for all patients, and this reduction was partly counteracted by the presence of MSCs as the AML cell viability was significantly increased for cytarabine-containing cultures with MSCs compared with corresponding cultures without MSCs. But the viability was still significantly lower than for drug-free direct cocultures (Figure 3B). Thus, the proapoptotic effect of cytarabine can be detected also in our *in vitro* model; this cytarabine effect can be partly counteracted by MSCs and taken together our overall results suggest that cytokine-mediated crosstalk between MSCs and AML cells can contribute to this effect.

### Effects of MSCs on H2AX Phosphorylation and mTOR Activation in Primary Human AML Cells

**Figure 4:**
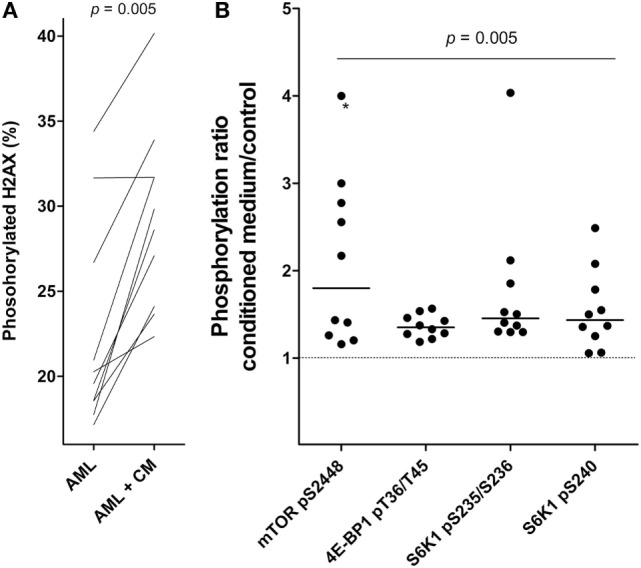
**Effects of mesenchymal stem cell (MSC)-conditioned medium on primary human acute myeloid leukemia (AML) cells; a study of H2AX phosphorylation and mTOR signaling**. **(A)** The change in percentage of phosphorylated H2AX for AML cells cultured in MSC-conditioned medium (CM, 50%) compared to medium alone. **(B)** The effect of MSC-conditioned medium on the phosphorylation of mTOR and its downstream targets. For each patient, we compared the level of phosphorylation for AML cells cultured with MSC-conditioned medium versus control cells cultured in medium alone. The results are presented as the relative level, i.e., the levels for MSC-conditioned cultures versus control cultures. The value marked with an asterisk represents a value that was undetectable in the control; for simplicity, the ratio was set to the same value as the highest observed ratio in the data series.

Phosphorylation of Histone H2AX can be seen as part of the DNA damage response and ATM activation; the phosphorylation can then be an early apoptotic event and has been used as an early marker of apoptosis induction (29). We performed cell-based ELISA prepared from cells cultured in MSC medium and in MSC24539-conditioned medium (50% final concentration; for the cytokine profile of this conditioned medium see Table 2). The percentage of phosphorylated H2AX showed a strong inverse correlation with AML cell viability for AML cells cultured in medium alone (Kendall’s tau; *p* < 0.0002), i.e., H2AX phosphorylation seems to be a marker of spontaneous *in vitro* apoptosis during culture of AML cells alone. However, significantly increased H2AX phosphorylation was observed after coculture with MSC-conditioned medium compared with leukemic cells cultured in medium alone (Figure 4A), and for these cultures, no significant association between viability and H2AX phosphorylation could be detected. This effect was also independent of AML cell proliferation and could be seen both for patients with increased proliferation in the presence of MSC-conditioned medium and patients with undetectable proliferation both with and without conditioned medium (data not shown). Thus, as will be discussed later, the increased H2AX phosphorylation in the presence of MSC-derived medium does not reflect increased apoptotic activity but rather a restoration of the decreased DNA damage response known to be present in primary human AML cells (30).

**Table 2:** **Cytokine excretion levels for 23 meditators from 51 acute myeloid leukemia (AML) blast supernatants (AML cells alone), AML blasts in coculture with mesenchymal stem cells (MSCs) (cocultures of AML cells and MSC24539) and only MSCs (MSC24539)**.

	AML cells alone			Cocultures of AML cells and MSC24539			MSC24539 alone	*p*-Value (supra-additive)
Mediator	# Patients	Median conc. (pg/mL)	Range (pg/mL)	# Patients	Median conc. (pg/mL)	Range (pg/mL)	Mean conc. (pg/mL)	
CXCL5	51	368	9.0–155,581	51	3,771	17–>213,550	25	<0.001
CXCL8	51	4,872	257–210,154	51	5,834	457–379,791	204	0.005
GM-CSF	50	3.5	n.d.–834	51	10	0.1–2,673	1.2	<0.001
IL-1RA	50	1,917	5.6–>25,500	50	1,562	5.6–>25,500	n.d.	n.s.
CCL5	50	56	n.d.–728	50	68	n.d.–17,161	2.9	n.s.
CXCL1	49	330	n.d.–35,641	51	11,316	24–42,042	32	<0.001
MMP-1	49	71	n.d.–14,263	51	18,223	157–>37,500	221	<0.001
CCL3	49	209	n.d.–41,519	51	234	8.0–42,167	12	<0.001
CCL4	48	235	n.d.–11,634	51	223	36–62,098	120	0.043
TNFα	45	6.0	n.d.–1,487	46	7.1	n.d.–10,676	0.8	0.037
bFGF	43	8.5	n.d.–57	50	19	n.d.–368	n.d.	<0.001
CCL2	51	992	9.2–22,859	51	3,888	613–20,621	2,643	n.s.
MMP-2	51	3,993	837–18,565	51	23,957	18,594–55,147	17,428	<0.001
CXCL10	39	7.1	n.d.–2,858	43	26	n.d.–2,882	n.d.	0.003
IL-6	39	14	n.d.–3,397	51	2,775	706–10,632	1,895	<0.001
CXCL11	37	4.1	n.d.–486	40	5.1	n.d.–377	1.9	0.014
IL-1β	29	3.5	n.d.–970	35	14	n.d.–21,593	n.d.	<0.001
IL-10	24	n.d.	n.d.–60	30	n.d.	0.8–601	n.d.	<0.001
IL-33	15	n.d.	n.d.–2.9	15	n.d.	n.d.–14	n.d.	n.s.
G-CSF	14	n.d.	n.d.–559	31	8.2	n.d.–>10,500	n.d.	<0.001
HGF	51	51	1.4–2,133	51	100	28–2,277	183	(0.001)
VEGF	50	17	n.d.–290	51	967	483–3,107	1,344	n.s.
Tie-2	45	50	n.d.–145	46	42	n.d.–298	14	(0.004)

*n.d., below detection limit; n.s., not significant; GM-CSF, granulocyte macrophage colony-stimulating factor; bFGF, basic fibroblastic growth factor; G-CSF, granulocyte colony-stimulating factor; HGF, hepatocyte growth factor; VEGF, vascular endothelial growth factor*.

*The soluble mediators are divided into three subgroups: cytokines that are released for a large majority of patients in both medium and coculture (above); mediators that are excreted for a subset of patients when leukemic cells were cultured in medium alone (middle); cytokines that are released to a significantly higher degree in MSCs than in AML cells alone or in coculture with MSCs, or with higher values in AML cells alone compared to cocultures. The background from the growth medium was subtracted from all values. *p*-Values (Wilcoxon test) for coculture values compared to additive levels of AML cells and MSCs are included on the right; significant *p*-values thus indicating a supra-additive effect. Values in brackets (HGF and Tie-2) indicate that coculture levels are significantly lower than the expected levels from secretion of AML cells and MSCs alone*.

We also investigated the effect of MSC-conditioned medium on Akt-mTOR signaling in primary human AML cells. An increased MSC-associated phosphorylation/activation of mTOR and its downstream targets, S6K1 and 4E-BP1, was then observed (Wilcoxon’s signed-rank test; *p* = 0.005; Figure 4B). The other upstream mediators did not differ significantly. Thus, MSCs alter H2AX phosphorylation as well as mTOR signaling in primary human AML cells, and as will be discussed later these effects may be important both for the MSC-associated growth enhancement and antiapoptotic effect of primary human AML cells.

### The Local Cytokine Network Is Altered during Coculture of Normal MSCs and Primary AML Cells

We determined the supernatant levels for 23 soluble mediators in transwell cocultures of MSC24539 and AML cells derived from all 51 patients (Table 2; Tables S2 and S3 in Supplementary Material). Our present study of this larger group confirmed the previous observations from a small group of 18 patients (16). First, the constitutive mediator release by primary AML cells shows a wide variation for each individual mediator, and this patient heterogeneity was maintained in cocultures as we observed significant correlations between the levels for AML cells cultured alone and the corresponding coculture for 22 of the 23 cytokines (Kendall’s tau correlation analysis), VEGF being the only exception. Second, relatively high levels were detected for most mediators in transwell cocultures both when compared with AML cells and MSC24539 cells cultured alone. Only two exceptional cytokines (HGF and VEGF, see Table 2) showed lower levels in the cocultures than in cultures of MSC24539 alone, and Tie-2 additionally showed higher levels in AML cell cultures than in cocultures. Third, supra-additive levels (i.e., cytokine levels in coculture supernatants exceeding the summarized levels for MSCs and AML cells cultured alone) were seen for several cytokines and reached statistical significance (Wilcoxon’s signed-rank tests, *p* ≤ 0.005 if not stated otherwise) when comparing the overall results for CCL3, CCL4 (*p* = 0.043), CXCL1, CXCL5, CXCL8, CXCL10, CXCL11 (*p* = 0.014), IL-1β, IL-6, IL-10, TNFα (*p* = 0.037), bFGF, G-CSF, GM-CSF, MMP-1, and MMP-2. Thus, even though individual differences among patients are maintained during coculture, several mediators show increased levels in cocultures and supra-additive levels are common.

**Figure 5:**
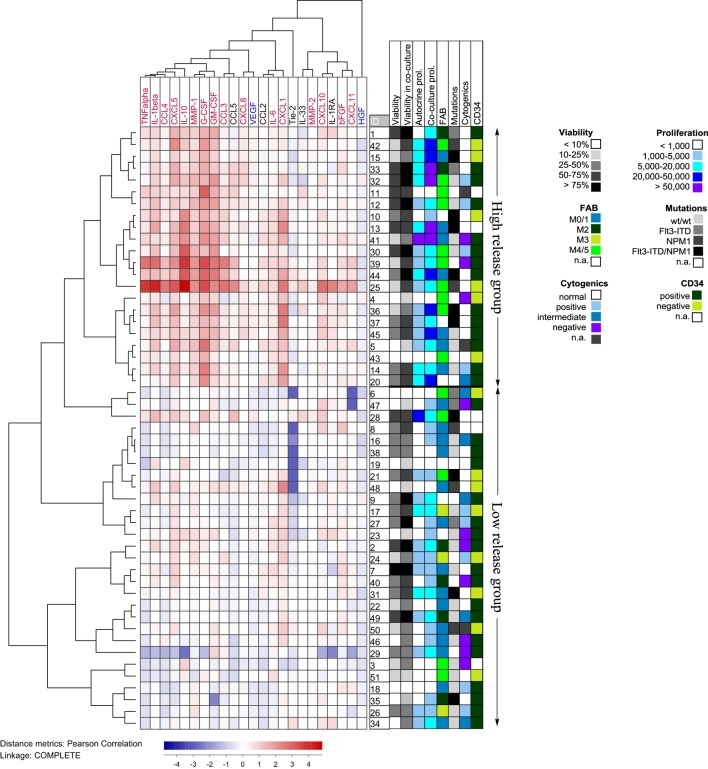
**Hierarchical clustering analysis based on the ratio of cytokine levels in acute myeloid leukemia (AML)-mesenchymal stem cell (MSC) cocultures divided by the concentration sums of AML cells and MSCs cultured alone; primary AML cells were derived from 51 patients and cocultured with MSC24539**. Each horizontal row in the figure represents the observation for one patient, and the vertical columns represent the observations for the soluble mediators. Red indicates supra-additive effects in coculture. Likewise cytokines marked in red showed supra-additive effect on the total patient cohort, whereas mediators marked in blue showed higher levels in MSC cultures alone than in coculture. The patients clustered into two main groups (see right part of the figure) indicating high and low relative coculture cytokine levels, respectively. The figure also shows the distribution of various biological characteristics between the patient subsets (AML cell viability after 72 h of culture in medium alone and in coculture, proliferative capacity in medium alone and in coculture, FAB classification, genetic abnormalities, and expression of the CD34 stem cell marker).

To further investigate the patient heterogeneity, we performed a cluster analysis (Figure 5). For each mediator and patient, we determined the ratio between the mediator level in coculture relative to the sum of the levels for MSC24539 and AML cells cultured alone. These ratios were log(10) transformed before the clustering; hence a value >0 indicates a supra-additive effect. The patients then separated into two main clusters: (i) one subgroup of 22 patients characterized by strong supra-additive effects for several cytokines, especially, CXCL1/5, IL-1β/10, TNFα, MMP-1, G-CSF, and GM-CSF and (ii) another patient subset with generally weaker effects. The patient subgroup with strong supra-additive effects was also (according to χ^2^ likelihood analyses) characterized by a significantly higher number of patients (17/22 versus 12/29, *p* = 0.009) showing detectable proliferation when cultured in the FBS-containing MSC medium alone without MSCs and also a higher number of patients (10/22 versus 0/29, *p* < 0.0001) with high AML cell proliferation exceeding >20,000 cpm in the cocultures. The supra-additive subset also showed a higher fraction of patients with cell viability >50% after coculture with MSCs (16/22 versus 11/29, *p* = 0.012). Finally, monocytic differentiation (FAB-M4/M5) was also more common among these patients (10/22 versus 5/29, *p* = 0.019). However, even though there was an association between supra-additive cytokine levels and high AML cell viability/proliferation in the cocultures, these supra-additive levels do not simply reflect increased proliferation/viability because supra-additive levels would then be expected for all the mediators and not only for a subset as we observed.

### The Cytokines Important for MSC-Mediated Growth Enhancement of AML Cells Differ among Patients

**Figure 6:**
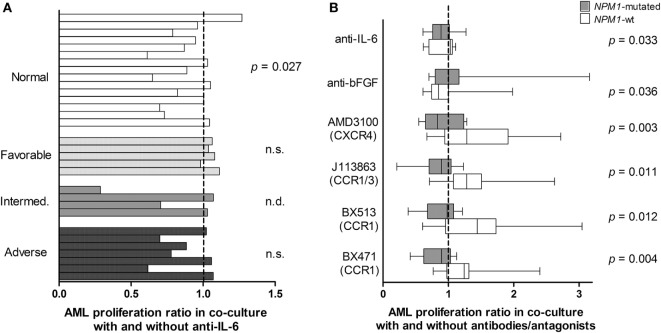
**Cytokine-targeted treatment during coculture of primary human acute myeloid leukemia (AML) cells and bone marrow mesenchymal stem cells (MSCs); a comparison of patients with different karyotype (A) and patients with and without *NPM1* mutations**. **(A)** Proliferative AML cell responses were compared for cocultures prepared with an IL-6 specific antibody and control antibody. The study included patients with normal AML cell karyotype (*n* = 15), favorable (*n* = 5), intermediate (*n* = 4), and adverse (*n* = 7) cytogenetics. The results for individual patients are presented as the proliferative response in cultures with anti-IL-6 relative to the proliferation in control cultures. The IL-6-specific antibody decreased the proliferation only for patients with normal cytogenetics but not for the other patients (Wilcoxon’s test for paired samples). **(B)** AML cell proliferation was also compared for patients with (gray columns) and without *NPM1* mutations (white columns). Decreased AML cell proliferation was seen for several cytokine-directed treatment, especially chemokine-directed interventions (Wilcoxon’s test for paired samples for IL-6/bFGF, χ^2^ likelihood analyses for the chemokines). The box-plots show the median value, the 25 and 75% quartiles and the variation range (minimum to maximum value).

To further study the cytokine-mediated crosstalk between MSCs and AML cells, we investigated the effects of cytokine-neutralizing antibodies and receptor antagonists on AML cell proliferation and viability in transwell cocultures with MSC24539. We then tested inhibitors of cytokines released by MSCs and being able to modulate AML cell proliferation (31–35), including (i) antibodies against VEGF, HGF, bFGF, and IL-6; (ii) the CCR1 antagonists BX471 (17) and BX513 (18); (iii) the combined CCR1 and CCR3 antagonist UCB35625 and its stereoisomer J113863 (19, 20); and (iv) the CXCR4 antagonist AMD3100 (13). The initial experiments included AML cells from eight patients that showed increased proliferation in cocultures corresponding to at least 3,000 cpm; this selection was made to be able to detect an inhibitory effect. Inhibition of AML cell proliferation for at least four patients was seen for anti-bFGF, anti-IL-6, the CCR1/CCR3 inhibitor J113863, and the CCR1 antagonists BX471 and BX513, whereas the CXCR4 inhibitor AMD3100 inhibited MSC24539 proliferation for four patients. These agents were further tested for 24 additional patients with an AML cell proliferation of at least 2,000 cpm in coculture. All but two of these patients then showed MSC-induced growth enhancement. When analyzing the overall results, none of the antibodies/inhibitors had any statistically significant effect, but the following observations were made for single mediators:

- *Normal karyotype*. The patients were heterogeneous with regard to karyotype. The only subset being large enough for statistical analysis was the 15 patients with normal karyotype, and for this subset, anti-IL-6 had a significant antiproliferative effect (Figure 6A, Wilcoxon’s test, *p* = 0.027) of borderline significance.
- *Patients with and without NPM1 mutations. NPM1* insertions were detected for 13 of the 32 patients, and all of them had normal karyotype. As would then be expected, anti-IL-6 had an antiproliferative effect for *NPM1*-mutated patients, but the statistical comparison reached only borderline significance (Wilcoxon’s test, *p* = 0.033). The same was true for anti-bFGF, which had an antiproliferative effect for patients with *NPM1*-wt (Figure 6B, Wilcoxon’s test, *p* = 0.036). Because several patients showed a relatively strong effect of chemokine-targeting pharmacological intervention we used statistical analysis based on categorized data when comparing *NPM1-*mutated and *NPM1*-wt patients. A significant alteration of AML cell proliferation was then defined as a difference having an absolute value of >2,000 cpm and in addition being >20% of the corresponding control. *NPM1*-wt was then associated with increased and *NPM1* mutation with decreased proliferation for all four chemokine receptor antagonists: AMD3100 (CXCR4 antagonist; χ^2^ likelihood analyses, *p* = 0.003), J113863 (CCR1/CCR3 antagonist, *p* = 0.011), BX513 (CCR1 antagonist, *p* = 0.012), and BX471 (CCR1 antagonist, *p* = 0.004). Thus, growth-modulating effects of several chemokines differed between *NPM1*-wt and *NPM1*-mutated patients.
- *FAB classification, CD34 expression, Flt3 mutations*. The effects of cytokine targeting showed significant associations neither with differentiation nor with *Flt3* mutations (data not shown).

**Figure 7:**
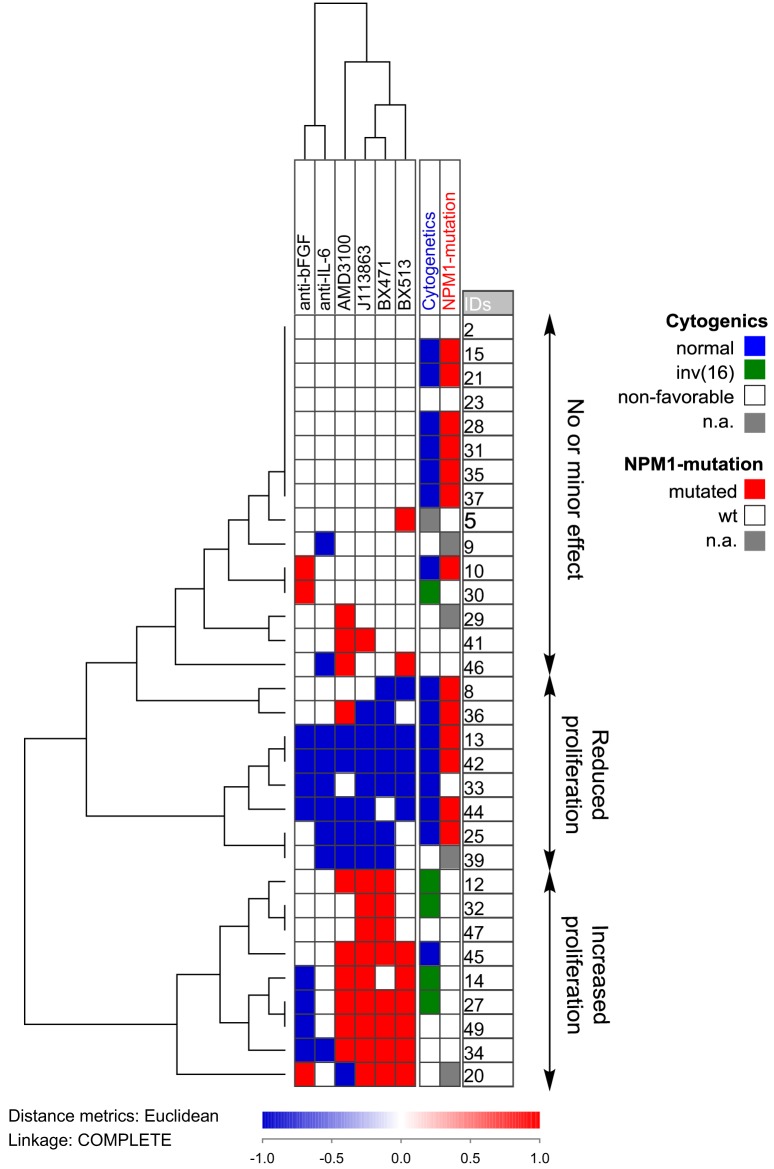
**Hierarchical clustering of the effects of anti-cytokine treatment (neutralizing antibodies or receptor blocking) on acute myeloid leukemia (AML) cell proliferation during coculture with normal mesenchymal stem cells**. AML cells derived from 32 AML patients were examined. Each horizontal row in the figure represents the observations for one patient, and the vertical columns represent the observations for each of the antibodies and receptor antagonists. Red indicates increased proliferation corresponding to an increase in the absolute value of >2,000 cpm and >20% proliferation increase compared to the control cultures, whereas blue indicates reduced proliferation according to the same definitions. The patients clustered into three main groups (see right part of the figure): one group (above) showing only minor effects, a second group (middle) showing reduced proliferation in the presence of both antibodies and receptor antagonists, and a last group (below) that showed increased proliferation especially for chemokine inhibition.

We also did a clustering analysis of the overall results (Figure 7) showing that especially the effect of chemokine inhibition differed among patients; growth reduction by CCR1/CXCR4 inhibition was seen especially for a subset of patients with normal cytogenetics and *NPM1* mutations. Based on this analysis, our patients could be classified into three different groups (no or minor effect, decreased proliferation, increased proliferation); these three groups did not significantly differ with regard to constitutive release of supra-additive coculture levels for IL-6, bFGF, or chemokines. Thus, these differences among patients are not caused by differences in cytokine release during coculture. Based on our overall results, we conclude that AML patients are heterogeneous also with regard to the effect (i.e., responsiveness) of single cytokines on AML cell proliferation in the presence of normal bone marrow MSCs, but despite this heterogeneity the final/overall cytokine-mediated MSC effect is increased AML cell proliferation for the majority of patients.

### Chemokine-Mediated Signaling Increases MSC Proliferation in the Presence of AML Cells

We further investigated the effects on MSC24539 proliferation of the same antibodies and antagonists as described above during coculture with AML cells from the same 32 patients. Anti-bFGF, anti-IL-6, and the CCR1 antagonist BX513 had only minimal effects (data not shown). In contrast, a significant antiproliferative effect was seen for the CXCR4 inhibitor AMD3100 (*p* < 0.0001), the CCR1/CCR3 inhibitor J113863 (*p* = 0.009), and the CCR1 antagonist BX417 (*p* = 0.015). Statistical analyses based on categorized data (i.e., increased, unaltered, decreased proliferation) showed associations between the CXCR4 mediated antiproliferative effect and monocytic AML cell differentiation (*p* = 0.009) and favorable karyotype (*p* = 0.025). These observations suggest that CCR1-, CXCR4-, and possibly CCR3-mediated signaling is important for the regulation of MSC proliferation in cocultures, and these results further illustrate that this bidirectional cytokine-mediated crosstalk between MSCs and AML cells affect not only the AML cells but also the MSCs.

## Discussion

Acute myeloid leukemia is a heterogeneous and aggressive malignancy; the long-term AML-free survival is only 50% even for younger patients who receive intensive chemotherapy and the majority of elderly patients who only receive leukemia-stabilizing treatment have a median survival of less than 1 year (36). Recent experimental studies suggest that both adhesion of AML cells to osteoblasts and MSCs (31, 37) and the release of soluble factors from the latter (38) are important for chemoresistance and thereby the risk of leukemia relapse from residual disease (35, 37). Malignant myeloid cells can also alter the functional characteristics of MSCs and thereby create a microenvironment that favors leukemic hematopoiesis (31, 39). Hence, therapeutic targeting of AML-supporting stromal cells may become a possible strategy to indirectly target the leukemia cells. In the present study, we therefore investigated the AML-supporting effects of bone marrow MSCs mediated through the local cytokine network for a large group of unselected leukemia patients.

Effects of stromal cells on primary human AML cells have also been investigated in previous studies (40–43), but none of these studies focused on the cytokine-mediated crosstalk between AML cells and MSCs. There were also several other differences: (i) only a low number of patients (41) or a low number of highly selected patients (42, 43) were examined; it is thereby difficult to address the question of patient heterogeneity; (ii) some of the studies used AML cell lines and not primary leukemic cells in parts of their experiments (42, 43); (iii) the previous studies used a stromal cell line (42, 43) or a single MSC donor (43). Thus, our present study extend the knowledge through its broader focus on the cytokine network and identification of mediators responsible for the leukemia-supporting effect of MSCs, studies of a large and unselected patient population, thereby addressing the question of patient heterogeneity, and the use of bone marrow MSCs derived from several donors.

Our viability assay has been described in detail in a previous publication (25). The AML cell population shows a hierarchical organization with a small number of leukemic stem cells, a minority of colony-forming proliferating cells and a majority of cells that shows spontaneous apoptosis during the first days of *in vitro* culture (25, 44). The number of viable cells will thereby decrease during culture and after four days of culture the viability will often be as low as 10–20% even for patients who show strong proliferative responses. This decreased viability during culture despite detectable proliferation shows that the viability of the total leukemic cell population mainly reflects the characteristics of the non-proliferating majority of AML cells and not the proliferation of a minor subset, i.e., survival and proliferation should be regarded as only partially overlapping characteristics. This is also supported by our recent results where a relatively small increase in AML cell viability during coculture was not exclusively seen for patients with undetectable proliferation but also for several other patients, and even patients with undetectable proliferation showed significantly increased viability (and several of them showed a relatively strong increase) after coculture with MSCs.

We used an experimental model based on transwell cocultures where MSCs and AML cells were separated by a semipermeable membrane; effects mediated through the bidirectional cytokine-mediated crosstalk could thereby be studied without any influence of additional effects mediated through direct cell contact. We used a culture medium supplemented by inactivated FBS and l-glutamine that is suitable for culture of both MSCs and primary AML cells (16). The MSCs then mediated antiapoptotic effects for all but three of the 51 AML patients included in the study. Furthermore, a large majority of patients (40 out of 51) showed growth enhancement in the presence of bone marrow MSCs, and this enhancement was seen both for short- and long-term AML cell proliferation, and for three different healthy MSC donors tested in independent experiments. The last 11 patients did not show detectable AML cell proliferation either in medium alone or during coculture with MSCs.

Primary AML cells show spontaneous or stress-induced apoptosis during *in vitro* culture, and our results demonstrated that MSCs can reduce this apoptosis. Our present results demonstrate that this antiapoptotic effect is also seen in the presence of cytarabine, i.e., the MSCs can also rescue primary AML cells from the combined effect of spontaneous and drug-induced apoptosis. However, we tested a cytarabine concentration that corresponds to systemic (i.e., serum) cytarabine levels reached during *in vivo* chemotherapy (50 nM), and in addition, a concentration corresponding to the steady-state levels during conventional cytarabine treatment with 100–200 mg/m^2^. A reduction of cell viability corresponding to more than 5% was seen only for approximately half of the patients when testing 50 nM cytarabine in the initial dose-response experiments in transwell cocultures, and in direct-contact cocultures. Both our experiments with transwell cocultures and cytarabine but especially the direct-contact experiments with 500 nM cytarabine showed that MSCs can counteract the proapoptotic effect of cytarabine, and at least for certain patients the cytokine-mediated crosstalk contributes to this effect. However, further studies are required to characterize and explain this patient heterogeneity and to clarify whether this variation is seen also for other chemotherapeutic agents.

The local cytokine network was also altered during AML-MSC coculture; the levels of three mediators were relatively low (Tie-2, HGF, and VEGF), whereas especially CXCL1/5, IL-1β/10, TNFα, MMP-1, G-CSF, and GM-CSF showed supra-additive effects in cocultures. A strong supra-additive effect for a subset of the mediators was especially seen for a patient subset also characterized by monocytic differentiation, high cell viability in coculture, and strong AML cell proliferation both when cultured in medium alone and in the cocultures with MSCs. The constitutive mediator release by AML cells cultured alone also differed considerably among patients, and despite the supra-additive effect for several cytokines, there was still a significant correlation for all but one mediator between the levels for AML cells cultured alone and in cocultures. Thus, individual differences between patients with regard to constitutive cytokine release by the AML cells are also maintained during coculture with MSCs.

We characterized the cytokine network in MSC/AML cell transwell cocultures in a previous study that also included a characterization of the effect of the AML-MSC crosstalk on the MSCs (16). The study included a smaller number of patients, but the effects of AML cells on the MSC showed only a limited variation among patients. Both MSCs and AML cells showed to contribute to the altered cytokine network during coculture, and the relative importance of MSCs and leukemic cells differed among cytokines. First, both primary human AML cells and bone marrow MSCs showed constitutive release of several cytokines, but the leukemic cells often showed a broader constitutive release profile than the MSCs (16). Second, the constitutive release by normal MSCs shows relatively small variations among individuals (16), whereas the constitutive leukemia cell release profile differed considerably among patients (16, 45). Third, cocultured MSCs showed increased mRNA expression of several cytokines, especially CCL and CXCL chemokines, as well as increased expression of several mediators of the cytokine-inducing NFκB pathway (16); signaling through this pathway induces increased constitutive release of several cytokines (45). Finally, there is a wide variation among patients in the constitutive release of several cytokines by their AML cells, and this variation is often maintained also in the presence of MSCs. Thus, the cytokine levels in our cocultures are probably determined by an NFκB-induced increase in the constitutive release by the MSCs and by maintenance of differences among patients in the constitutive release by the AML cells.

We investigated possible molecular mechanisms behind the growth-enhancing and antiapoptotic effect of MSCs on primary human AML cells. These experiments included leukemic cells from 10 patients. The PI3K–Akt–mTOR pathway is often constitutively activated in AML cells and is important for cell survival, proliferation and metabolism (46, 47). We therefore compared the activation/phosphorylation of Akt, mTOR, and mediators downstream to mTOR (S6K1, 4E-BP1) after incubation with medium alone or 50% MSC-conditioned medium. Akt phosphorylation did not differ among patients, whereas increased phosphorylation was detected for mTOR and its downstream mediators.

We also compared the effect of MSC-conditioned medium on the phosphorylation of the H2AX histone that is phosphorylated by the ATM kinase as part of the DNA damage response. There will be a background or constitutive level of H2AX phosphorylation, and this level seems to reflect a DNA damage response initiated by endogenous formation of reactive oxidant species as a byproduct from oxidative phosphorylation (48). However, DNA damage response with increased H2AX phosphorylation can also be an early event during apoptosis (29). Primary human AML cells seem to have a suppressed DNA damage response due to increased expression of double-stranded RNA-activated protein kinase, and high expression of this kinase in the AML cells is thereby associated with an inhibited DNA damage response reflected as a low percentage of H2AX phosphorylation (49, 50). H2AX phosphorylation can also be seen without DNA damage (51). In our experiments we observed a significant increase in H2AX phosphorylation after culture with MSC-conditioned medium (Wilcoxon’s test; *p* = 0.005). However, this increase of H2AX phosphorylation combined with increased cell viability suggests that the increased H2AX phosphorylation is not an effect of increased apoptosis. In our opinion, the most likely explanation is a reversal of the AML-associated decrease in the DNA damage response; this may be caused by IL-6 that is present at high levels in MSC-conditioned medium (see Table 2) and which is known to strengthen the DNA damage response (30). An alternative explanation could be that increased mTOR mediated signaling causes increased oxidative phosphorylation and thereby increased constitutive (i.e., oxidative) DNA damage (52). Thus, both increased mTOR activation and increased DNA damage responsiveness can contribute to the effects of MSCs on primary human AML cells.

Finally, cytokine neutralization and receptor blocking showed that AML cells derived from different patients were heterogeneous with regard to the effects of various cytokines on AML cell viability/proliferation. One of the cytokines with differential effects was CXCL12, which is highly released by MSCs. Even though cells with *Flt3*-ITD have shown increased CXCR4 expression (13) and signaling through the CXCL12-CXCR4 axis, the differential effect of CXCR4 blocking showed no significant association with *Flt3*-ITD.

Several soluble mediators that were detected at relatively high levels in our cocultures have been linked with remodeling of the bone marrow stem cell niche into a leukemia-permissive niche: (i) angiogenic growth factors like VEGF (53, 54), bFGF, Ang-1, and its receptor Tie-2 (31); (ii) IL-6 (32), which also can upregulate VEGF levels (55); (iii) CCL3, which is thought to expand MSCs and drive them into a leukemia-supporting phenotype (39); (iv) CXCL12, which is constitutively expressed by MSCs (37), causes homing of leukemic cells to the bone marrow (56, 57) and functions as a regulator of proliferation, cell cycle progression, and survival of leukemic cells (35, 58). Our previous studies could not detect any evidence for osteoblastic or adipocytic MSC differentiation during MSC/AML cell cocultures, even though the global gene expression profile of normal MSCs was seen after coculture of MSC and AML cells in transwell cultures (16).

MSCs showed constitutive release of several mediators, and we used cytokine-neutralizing antibodies or receptor-blocking agents to identify cytokines that contributed to the antiapoptotic and growth-enhancing effect of MSCs on the AML cells. We then investigated cytokines that were released by MSCs at relatively high levels, showed high levels during coculture and are known to function as growth factors for primary human AML cells. First, antibodies against HGF and VEGF tested separately had no or only minimal effects on AML cell proliferation and were only examined in the initial experiments. Second, IL-6 neutralization inhibited AML cell proliferation for the subset of patients with normal cytogenetics, whereas reduced proliferation upon bFGF neutralization was only observed in *NPM1-*wt cells and this is consistent with previous observations of AML cells cultured alone (33). Third, the effects of several chemokine receptor blockers (including CXCL12/CXCR4 blocking) also differed among patients, especially when comparing patients with and without *NPM1* mutations. Based on the overall results, we conclude that AML patients are heterogeneous with regard to effects of individual cytokines on AML cell viability and proliferation during MSC/AML cells coculture. However, despite this variation, the final overall cytokine-mediated effects of the MSCs are increased viability and growth enhancement probably caused by a combined effect of several cytokines, and the cytokines contributing to this effect seem to differ among patients.

In contrast to the divergent effects of cytokine neutralization/blocking on AML cells, these interventions had more uniform effects on MSC proliferation during coculture as MSC proliferation significantly decreased in response to chemokine receptor blocking (CCR1, CCR3, CXCR4) agents. Both AML cells and MSCs show constitutive release of several ligands for these receptors, suggesting that autocrine or paracrine loops involving these receptors are important for the regulation of MSC proliferation (59). This hypothesis is also supported by the observation that CCL3 expression by malignant myeloid cells is linked with higher MSC growth rates (39).

Systemic plasma levels of both IL-6, bFGF, and several chemokines have been investigated in human AML. IL-6 levels are increased in patients with untreated AML and high levels seem to be associated with decreased survival (60). In contrast, the results for plasma bFGF levels are conflicting and both normal and increased plasma levels have been described for patients with untreated AML, but none of these studies have described any prognostic impact of bFGF levels (61–63). As reviewed by Reikvam et al. (64), several studies have investigated the systemic (plasma or serum) levels of various chemokines; besides normal, both increased and decreased levels have been described for most of the investigated CCL and CXCL chemokines in patients with untreated AML. Even though only a small minority of patients (<15%) shows detectable CXCL12 release during *in vitro* culture and most of these patients show only low release (45), increased serum CXCL12 levels have been described in patients with untreated AML, including increased levels of the cleaved active form. These observations may suggest that constitutive AML cell release of these cytokines has clinical relevance in human AML. However, the observations should be interpreted with great care because these cytokines can be released by several normal cells and not only AML cells, some of these cytokines may be a part of the acute phase reaction, and the systemic levels reflect the binding between release and binding/degradation, Thus, altered systemic levels may not reflect AML cell release or the local levels in the bone marrow microenvironment.

## Conclusion

Our present study shows that even though AML is a heterogeneous disease and the response of primary AML cells to the various MSC-derived cytokines during MSC-AML cell coculture differs among patients, the final effect of MSC-derived cytokines on primary AML cells is increased proliferation and viability. Our overall results suggest that therapeutic targeting of the cytokine-mediated AML-supporting effects probably as MSC-directed strategies which inhibit the release of several cytokines, or alternatively receptor blocking could be tried in selected patients.

## Author Contributions

ØB designed the study and wrote the paper. IN performed the phosphoflow experiments and analyzed the results. AB performed the other experiments, analyzed the results, and wrote the paper.

## Conflict of Interest Statement

The authors declare that the research was conducted in the absence of any commercial or financial relationships that could be construed as a potential conflict of interest.

## Abbreviations

AML, acute myeloid leukemia; Ang-1, angiopoietin 1; ATM, ataxia telangiectasia, mutated; bFGF, basic fibroblastic growth factor; CFU, colony-forming unit; cpm, counts per minute; ELISA, enzyme-linked immunosorbent assay; EPO, erythropoietin; FBS, fetal bovine serum; Flt3, FMS-related tyrosine kinase 3; G-CSF, granulocyte colony stimulating factor; GM-CSF, granulocyte macrophage colony-stimulating factor; HGF, hepatocyte growth factor; MMP, matrix metalloproteinase; MSC, mesenchymal stem cell; MSCGM, mesenchymal stem cell growth medium; NPM1, nucleophosmin; PI, propidium iodide; TNFα, tumor necrosis factor α; VEGF, vascular endothelial growth factor.

## References

[1] Estey EH. Acute myeloid leukemia: 2014 update on risk-stratification and management. Am J Hematol (2014) 89:1063–81. 10.1002/ajh.23834 25318680

[2] Arber DA Orazi A Hasserjian R Thiele J Borowitz MJ Le Beau MM The 2016 revision to the World Health Organization classification of myeloid neoplasms and acute leukemia. Blood (2016) 127:2391–405. 10.1182/blood-2016-03-643544 27069254

[3] Burnett A Wetzler M Löwenberg B. Therapeutic advances in acute myeloid leukemia. J Clin Oncol (2011) 29:487–94. 10.1200/JCO.2010.30.1820 21220605

[4] Falini B Nicoletti I Bolli N Martelli MP Liso A Gorello P Translocations and mutations involving the nucleophosmin (NPM1) gene in lymphomas and leukemias. Haematologica (2007) 92:519–32. 10.3324/haematol.11007 17488663

[5] Deans RJ Moseley AB. Mesenchymal stem cells: biology and potential clinical uses. Exp Hematol (2000) 28:875–84. 10.1016/S0301-472X(00)00482-3 10989188

[6] Lv FJ Tuan RS Cheung KM Leung VY The surface markers and identity of human mesenchymal stem cells. Stem Cells (2014) 32:1408–19. 10.1002/stem.1681 24578244

[7] Jiang Y Jahagirdar BN Reinhardt RL Schwartz RE Keene CD Ortiz-Gonzalez XR Pluripotency of mesenchymal stem cells derived from adult marrow. Nature (2002) 418:41–9. 10.1038/nature00870 12077603

[8] Eggenhofer E Luk F Dahlke MH Hoogduijn MJ. The life and fate of mesenchymal stem cells. Front Immunol (2014) 5:148. 10.3389/fimmu.2014.00148 24904568 PMC4032901

[9] Uccelli A Moretta L Pistoia V. Mesenchymal stem cells in health and disease. Nat Rev Immunol (2008) 8:726–36. 10.1038/nri2395 19172693

[10] Morandi F Raffaghello L Bianchi G Meloni F Salis A Millo E Immunogenicity of human mesenchymal stem cells in HLA-class I-restricted T-cell responses against viral or tumor-associated antigens. Stem Cells (2008) 26:1275–87. 10.1634/stemcells.2007-0878 18292209 PMC2726787

[11] Lane SW Scadden DT Gilliland DG. The leukemic stem cell niche: current concepts and therapeutic opportunities. Blood (2009) 114:1150–7. 10.1182/blood-2009-01-202606 19401558 PMC2723012

[12] Blau O Baldus CD Hofmann WK Thiel G Nolte F Burmeister T Mesenchymal stromal cells of myelodysplastic syndrome and acute myeloid leukemia patients have distinct genetic abnormalities compared with leukemic blasts. Blood (2011) 118:5583–92. 10.1182/blood-2011-03-343467 21948175 PMC3217359

[13] Kojima K McQueen T Chen Y Jacamo R Konopleva M Shinojima N p53 activation of mesenchymal stromal cells partially abrogates microenvironment-mediated resistance to FLT3 inhibition in AML through HIF-1alpha-mediated down-regulation of CXCL12. Blood (2011) 118:4431–9. 10.1182/blood-2011-02-334136 21868571 PMC3204912

[14] Reikvam H Hatfield KJ Fredly H Nepstad I Mosevoll KA Bruserud O. The angioregulatory cytokine network in human acute myeloid leukemia – from leukemogenesis via remission induction to stem cell transplantation. Eur Cytokine Netw (2012) 23:140–53. 10.1684/ecn.2012.0322 23328436

[15] Bruserud Ø Ryningen A Wergeland L Glenjen NI Gjertsen BT. Osteoblasts increase proliferation and release of pro-angiogenic interleukin 8 by native human acute myelogenous leukemia blasts. Haematologica (2004) 89:391–402. 15075072

[16] Reikvam H Brenner AK Hagen KM Liseth K Skrede S Hatfield KJ The cytokine-mediated crosstalk between primary human acute myeloid cells and mesenchymal stem cells alters the local cytokine network and the global gene expression profile of the mesenchymal cells. Stem Cell Res (2015) 15:530–41. 10.1016/j.scr.2015.09.008 26468600

[17] Menu E De Leenheer E De Raeve H Coulton L Imanishi T Miyashita K Role of CCR1 and CCR5 in homing and growth of multiple myeloma and in the development of osteolytic lesions: a study in the 5TMM model. Clin Exp Metastasis (2006) 23:291–300. 10.1007/s10585-006-9038-6 17086356

[18] Di Prisco S Merega E Pittaluga A. Functional adaptation of presynaptic chemokine receptors in EAE mouse central nervous system. Synapse (2014) 68:529–35. 10.1002/syn.21774 25092801

[19] de Mendonca FL da Fonseca PC Phillips RM Saldanha JW Williams TJ Pease JE. Site-directed mutagenesis of CC chemokine receptor 1 reveals the mechanism of action of UCB 35625, a small molecule chemokine receptor antagonist. J Biol Chem (2005) 280:4808–16. 10.1074/jbc.M412267200 15548526

[20] Sabroe I Peck MJ Van Keulen BJ Jorritsma A Simmons G Clapham PR A small molecule antagonist of chemokine receptors CCR1 and CCR3. Potent inhibition of eosinophil function and CCR3-mediated HIV-1 entry. J Biol Chem (2000) 275:25985–92. 10.1074/jbc.M908864199 10854442

[21] Ersvaer E Brenner AK Vetas K Reikvam H Bruserud Ø. Effects of cytarabine on activation of human T cells – cytarabine has concentration-dependent effects that are modulated both by valproic acid and all-trans retinoic acid. BMC Pharmacol Toxicol (2015) 16:12. 10.1186/s40360-015-0012-2 25934555 PMC4422044

[22] Reikvam H Øyan AM Kalland KH Hovland R Hatfield KJ Bruserud Ø. Differences in proliferative capacity of primary human acute myelogenous leukaemia cells are associated with altered gene expression profiles and can be used for subclassification of patients. Cell Prolif (2013) 46:554–62. 10.1111/cpr.12057 24073609 PMC6495661

[23] Vermes I Haanen C Steffensnakken H Reutelingsperger C A novel assay for apoptosis – flow cytometric detection of phosphatidylserine expression on early apoptotic cells using fluorescein-labeled annexin-V. J Immunol Methods (1995) 184:39–51. 10.1016/0022-1759(95)00072-I 7622868

[24] Skavland J Jorgensen KM Hadziavdic K Hovland R Jonassen I Bruserud Ø Specific cellular signal-transduction responses to in vivo combination therapy with ATRA, valproic acid and theophylline in acute myeloid leukemia. Blood Cancer J (2011) 1:e4. 10.1038/bcj.2011.2 22829110 PMC3255270

[25] Ryningen A Ersvaer E Øyan AM Kalland KH Vintermyr OK Gjertsen BT Stress-induced in vitro apoptosis of native human acute myelogenous leukemia (AML) cells shows a wide variation between patients and is associated with low BCL-2:Bax ratio and low levels of heat shock protein 70 and 90. Leuk Res (2006) 30:1531–40. 10.1016/j.leukres.2006.02.014 16600371

[26] Griessinger E Anjos-Afonso F Pizzitola I Rouault-Pierre K Vargaftig J Taussig D A niche-like culture system allowing the maintenance of primary human acute myeloid leukemia-initiating cells: a new tool to decipher their chemoresistance and self-renewal mechanisms. Stem Cells Transl Med (2014) 3:520–9. 10.5966/sctm.2013-0166 24493855 PMC3973718

[27] Hubeek I Kaspers G Ossenkoppele G Peters G Deoxynucleoside analogs in cancer therapy. Cancer Drug Discovery and Development (2006). p. 119–52.

[28] Fredly H Ersvaer E Kittang AO Tsykunova G Gjertsen BT Bruserud Ø. The combination of valproic acid, all-trans retinoic acid and low-dose cytarabine as disease-stabilizing treatment in acute myeloid leukemia. Clin Epigenetics (2013) 5:13. 10.1186/1868-7083-5-13 23915396 PMC3765924

[29] Tanaka T Huang X Halicka HD Zhao H Traganos F Albino AP Cytometry of ATM activation and histone H2AX phosphorylation to estimate extent of DNA damage induced by exogenous agents. Cytometry A (2007) 71:648–61. 10.1002/cyto.a.20426 17622968 PMC3855668

[30] Centurione L Aiello FB DNA repair and cytokines: TGF-beta, IL-6, and thrombopoietin as different biomarkers of radioresistance. Front Oncol (2016) 6:175 10.3389/fonc.2016.00175 27500125 PMC4956642

[31] Ghiaur G Wroblewski M Loges S. Acute myelogenous leukemia and its microenvironment: a molecular conversation. Semin Hematol (2015) 52:200–6. 10.1053/j.seminhematol.2015.03.003 26111467

[32] Golay J Cuppini L Leoni F Mico C Barbui V Domenghini M The histone deacetylase inhibitor ITF2357 has anti-leukemic activity in vitro and in vivo and inhibits IL-6 and VEGF production by stromal cells. Leukemia (2007) 21:1892–900. 10.1038/sj.leu.2404860 17637810

[33] Karajannis MA Vincent L Direnzo R Shmelkov SV Zhang F Feldman EJ Activation of FGFR1beta signaling pathway promotes survival, migration and resistance to chemotherapy in acute myeloid leukemia cells. Leukemia (2006) 20:979–86. 10.1038/sj.leu.2404203 16598308

[34] Oba Y Lee JW Ehrlich LA Chung HY Jelinek DF Callander NS MIP-1alpha utilizes both CCR1 and CCR5 to induce osteoclast formation and increase adhesion of myeloma cells to marrow stromal cells. Exp Hematol (2005) 33:272–8. 10.1016/j.exphem.2004.11.015 15730850

[35] Tavor S Eisenbach M Jacob-Hirsch J Golan T Petit I Benzion K The CXCR4 antagonist AMD3100 impairs survival of human AML cells and induces their differentiation. Leukemia (2008) 22:2151–5158. 10.1038/leu.2008.238 18769446

[36] Estey E Döhner H. Acute myeloid leukaemia. Lancet (2006) 368:1894–907. 10.1016/S0140-6736(06)69780-8 17126723

[37] Burger JA Bürkle A. The CXCR4 chemokine receptor in acute and chronic leukaemia: a marrow homing receptor and potential therapeutic target. Br J Haematol (2007) 137:288–96. 10.1111/j.1365-2141.2007.06590.x 17456052

[38] Nervi B Ramirez P Rettig MP Uy GL Holt MS Ritchey JK Chemosensitization of acute myeloid leukemia (AML) following mobilization by the CXCR4 antagonist AMD3100. Blood (2009) 113:6206–14. 10.1182/blood-2008-06-162123 19050309 PMC2699239

[39] Schepers K Pietras EM Reynaud D Flach J Binnewies M Garg T Myeloproliferative neoplasia remodels the endosteal bone marrow niche into a self-reinforcing leukemic niche. Cell Stem Cell (2013) 13:285–99. 10.1016/j.stem.2013.06.009 23850243 PMC3769504

[40] Garrido SM Appelbaum FR Willman CL Banker DE Acute myeloid leukemia cells are protected from spontaneous and drug-induced apoptosis by direct contact with a human bone marrow stromal cell line (HS-5). Exp Hematol (2001) 29:448–57. 10.1016/S0301-472X(01)00612-9 11301185

[41] Ito S Barrett AJ Dutra A Pak E Miner S Keyvanfar K Long term maintenance of myeloid leukemic stem cells cultured with unrelated human mesenchymal stromal cells. Stem Cell Res (2015) 14:95–104. 10.1016/j.scr.2014.11.007 25535865 PMC4634876

[42] Konopleva M Konoplev S Hu W Zaritskey AY Afanasiev BV Andreeff M. Stromal cells prevent apoptosis of AML cells by up-regulation of anti-apoptotic proteins. Leukemia (2002) 16:1713–24. 10.1038/sj.leu.2402608 12200686

[43] Tabe Y Jin L Tsutsumi-Ishii Y Xu Y McQueen T Priebe W Activation of integrin-linked kinase is a critical prosurvival pathway induced in leukemic cells by bone marrow-derived stromal cells. Cancer Res (2007) 67:684–94. 10.1158/0008-5472.CAN-06-3166 17234779

[44] Bruserud Ø Gjertsen BT Foss B Huang TS New strategies in the treatment of acute myelogenous leukemia (AML): in vitro culture of aml cells – the present use in experimental studies and the possible importance for future therapeutic approaches. Stem Cells (2001) 19:1–11. 10.1634/stemcells.19-1-1 11209086

[45] Bruserud Ø Ryningen A Olsnes AM Stordrange L Øyan AM Kalland KH Subclassification of patients with acute myelogenous leukemia based on chemokine responsiveness and constitutive chemokine release by their leukemic cells. Haematologica (2007) 92:332–41. 10.3324/haematol.10148 17339182

[46] Altman JK Sassano A Platanias LC. Targeting mTOR for the treatment of AML. New agents and new directions. Oncotarget (2011) 2:510–7. 10.18632/oncotarget.290 21680954 PMC3248202

[47] Park S Chapuis N Tamburini J Bardet V Cornillet-Lefebvre P Willems L Role of the PI3K/AKT and mTOR signaling pathways in acute myeloid leukemia. Haematologica (2010) 95:819–28. 10.3324/haematol.2009.013797 19951971 PMC2864389

[48] Huang X Tanaka T Kurose A Traganos F Darzynkiewicz Z. Constitutive histone H2AX phosphorylation on Ser-139 in cells untreated by genotoxic agents is cell-cycle phase specific and attenuated by scavenging reactive oxygen species. Int J Oncol (2006) 29:495–501. 10.3892/ijo.29.2.495 16820894

[49] Boehrer S Ades L Tajeddine N Hofmann WK Kriener S Bug G Suppression of the DNA damage response in acute myeloid leukemia versus myelodysplastic syndrome. Oncogene (2009) 28:2205–18. 10.1038/onc.2009.69 19398952

[50] Cheng X Byrne M Brown KD Konopleva MY Kornblau SM Bennett RL PKR inhibits the DNA damage response, and is associated with poor survival in AML and accelerated leukemia in NHD13 mice. Blood (2015) 126:1585–94. 10.1182/blood-2015-03-635227 26202421 PMC4582335

[51] Ichijima Y Sakasai R Okita N Asahina K Mizutani S Teraoka H. Phosphorylation of histone H2AX at M phase in human cells without DNA damage response. Biochem Biophys Res Commun (2005) 336:807–12. 10.1016/j.bbrc.2005.08.164 16153602

[52] Darzynkiewicz Z Zhao H Halicka HD Li J Lee YS Hsieh TC In search of antiaging modalities: evaluation of mTOR- and ROS/DNA damage-signaling by cytometry. Cytometry A (2014) 85:386–99. 10.1002/cyto.a.22452 24677687 PMC4080725

[53] Imai N Miwa H Shikami M Suganuma K Gotoh M Hiramatsu A Growth inhibition of AML cells with specific chromosome abnormalities by monoclonal antibodies to receptors for vascular endothelial growth factor. Leuk Res (2009) 33:1650–7. 10.1016/j.leukres.2009.03.006 19342098

[54] Kampen KR Ter Elst A de Bont ES. Vascular endothelial growth factor signaling in acute myeloid leukemia. Cell Mol Life Sci (2013) 70:1307–17. 10.1007/s00018-012-1085-3 22833169 PMC11113417

[55] Yao X Huang J Zhong H Shen N Faggioni R Fung M Targeting interleukin-6 in inflammatory autoimmune diseases and cancers. Pharmacol Ther (2014) 141:125–39. 10.1016/j.pharmthera.2013.09.004 24076269

[56] Alfano D Gorrasi A Li Santi A Ricci P Montuori N Selleri C Urokinase receptor and CXCR4 are regulated by common microRNAs in leukaemia cells. J Cell Mol Med (2015) 19(9):2262–72. 10.1111/jcmm.12617 26082201 PMC4568930

[57] Jacobi A Thieme S Lehmann R Ugarte F Malech HL Koch S Impact of CXCR4 inhibition on FLT3-ITD-positive human AML blasts. Exp Hematol (2010) 38:180–90. 10.1016/j.exphem.2009.12.003 20035824 PMC4777334

[58] Spinello I Quaranta MT Riccioni R Riti V Pasquini L Boe A MicroRNA-146a and AMD3100, two ways to control CXCR4 expression in acute myeloid leukemias. Blood Cancer J (2011) 1:e26. 10.1038/bcj.2011.24 22829170 PMC3255264

[59] Vallet S Anderson KC. CCR1 as a target for multiple myeloma. Expert Opin Ther Targets (2011) 15:1037–47. 10.1517/14728222.2011.586634 21609295

[60] Sanchez-Correa B Bergua JM Campos C Gayoso I Arcos MJ Banas H Cytokine profiles in acute myeloid leukemia patients at diagnosis: survival is inversely correlated with IL-6 and directly correlated with IL-10 levels. Cytokine (2013) 61:885–91. 10.1016/j.cyto.2012.12.023 23357299

[61] Aguayo A Kantarjian H Manshouri T Gidel C Estey E Thomas D Angiogenesis in acute and chronic leukemias and myelodysplastic syndromes. Blood (2000) 96:2240–5. 10979972

[62] Aguayo A Kantarjian HM Estey EH Giles FJ Verstovsek S Manshouri T Plasma vascular endothelial growth factor levels have prognostic significance in patients with acute myeloid leukemia but not in patients with myelodysplastic syndromes. Cancer (2002) 95:1923–30. 10.1002/cncr.10900 12404286

[63] Brunner B Gunsilius E Schumacher P Zwierzina H Gastl G Stauder R. Blood levels of angiogenin and vascular endothelial growth factor are elevated in myelodysplastic syndromes and in acute myeloid leukemia. J Hematother Stem Cell Res (2002) 11:119–25. 10.1089/152581602753448586 11847008

[64] Reikvam H Fredly H Kittang AO Bruserud Ø The possible diagnostic and prognostic use of systemic chemokine profiles in clinical medicine-the experience in acute myeloid leukemia from disease development and diagnosis via conventional chemotherapy to allogeneic stem cell transplantation. Toxins (Basel) (2013) 5:336–62. 10.3390/toxins5020336 23430540 PMC3640539

