# Supplementary material for: Mesenchymal Stem Cells Support Survival and Proliferation of Primary Human Acute Myeloid Leukemia Cells through Heterogeneous Molecular Mechanisms
Source: Front Immunol. 2017 Feb 9;8:106. doi: 10.3389/fimmu.2017.00106 (PMC5299032; doi:10.3389/fimmu.2017.00106)
Supplement: Supplementary file 1 [file Data_Sheet_1.PDF]

Supplementary data

MESENCHYMAL STEM CELLS SUPPORT SURVIVAL AND PROLIFERATION OF PRIMARY HUMAN ACUTE MYELOID LEUKEMIA CELLS THROUGH HETEROGENEOUS MOLECULAR MECHANISMS

Annette K. Brenner [1,*], Ina Nepstad [1], Øystein Bruserud [1,2]

[1]Section for Hematology, Department of Clinical Science, University of Bergen, Bergen, Norway

[2] Department of Medicine, Haukeland University Hospital; Bergen, Norway

Supplementary Table 1 Effects of MSCs on AML cell proliferation and viability - a summary of the overall results when testing normal bone marrow MSCs derived from 3 different donors and primary human AML cells derived from 18 unselected patients.

	**MSC24429**		**MSC24539**		**MSC25200**	
	AML cells alone	Co-culture	AML cells alone	Co-culture	AML cells alone	Co-culture
**AML cell proliferation**						
Number of patients with AML cell proliferation > 1,000 cpm	7	15	12	15	8	13
Median (cpm)	< 1,000	3,316	3,693	14,187	< 1,000	3,517
Lowest response > 1,000 cpm	1,130	1,145	1,597	2,247	1,046	1,128
Highest response (cpm)	11,398	30,535	25,887	87,105	10,238	26,378
**AML cell viability**						
Median (%)	11.6	23.1	29.5	55.8	13.0	28.5
Lowest value (%)	1.1	2.5	3.3	10.5	0.4	0.8
Highest value (%)	84.7	97.7	70.9	93.8	57.9	81.7

Supplementary Table 2 AML cytokine excretion levels for four meditators that are affected by antibody- and antagonist inhibition (bFGF, IL-6, CCL3 and CCL5) for patients with normal, favorable, intermediate and adverse cytogenetics. There were no significant differences in these values among the four cytogenetic groups (Kruskal-Wallis test).

	Normal			Favorable			Intermediate			Adverse		
**Mediator**	# patients[1]	Median conc. (pg/mL)	Range (pg/mL)	# patients	Median conc. (pg/mL)	Range (pg/mL)	# patients	Median conc. (pg/mL)	Range (pg/mL)	# patients	Median conc. (pg/mL)	Range (pg/mL)
CCL3	23	272	2.7 – 41,519	8	394	n.d. – 20,841	5	117	n.d. – 534	10	171	13 – 2,869
CCL5	23	66	6.4 – 414	9	60	16 – 728	5	53	n.d. – 119	10	50	6.4 – 369
bFGF	17	8.5	n.d[2]. – 46	8	8.5	n.d. – 57	5	11	n.d. – 19	10	14	4.3 – 50
IL-6	19	39	0.7 – 2,775	6	6.8	n.d. – 1,501	1	n.d.	n.d. – 2.1	9	20	n.d. – 3,397

[1]patients with detectable excretion levels
[2]below detection limit

Supplementary Table 3 AML cytokine excretion levels for four meditators that are affected by antibody- and antagonist inhibition (bFGF, IL-6, CCL3 and CCL5) for patients with and without *NPM1*-mutations. The secretion levels of these mediators did not differ significantly between the two patient groups (Mann-Whitney *U*-test).

	***NPM1*-insertion**			***NPM1*-wt**		
**Mediator**	# patients[1]	Median conc. (pg/mL)	Range (pg/mL)	# patients	Median conc. (pg/mL)	Range (pg/mL)
CCL3	16	320	18 – 41,519	24	172	n.d. – 20,841
CCL5	16	85	6.4 – 414	25	47	n.d. – 728
bFGF	14	8.5	n.d.[2] – 28	21	8.5	n.d. – 57
IL-6	14	26	n.d. – 2,775	16	5.4	n.d. – 1,501

[1]patients with detectable excretion levels

[2]below detection limit

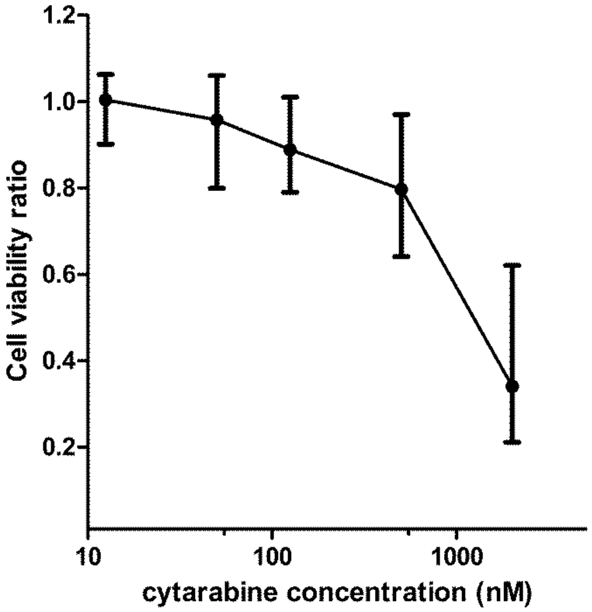

Supplementary Figure 1 Primary human AML cells were cultured in microtiter cultures and viability assayed by flow cytometry as described in detail in a previous article (1). Viability was assayed by flow cytometry and for each culture 20,000 cells were analyzed. The cells were cryopreserved and the viability immediately after thawing (before culture) corresponded to 73-80 % viable cells. The cells were cultured for 40 hours and the cell viability in drug-free controls then corresponded to median 43 % (range 21.6 – 71.1%); this reduction in cell viability during culture is caused by spontaneous or drug-induced apoptosis that causes a gradual reduction of AML cell viability during culture (2, 3). We investigated the effect of cytarabine 12.5, 50, 125, 500 and 2000 nM on cell viability; the results are presented as the median with the 25 % and 75 % percentiles. As can be seen from the figure, the effect of cytarabine 50-500 nM showed a large overlap and differed among patients. However, a reduced viability was seen for a large subset of patients when testing these concentrations, i.e. for 50 nM and 500 nM a decrease in viable cells of at least 5 % and 20 %, respectively, was seen for half of the tested patients. Based on these experiments, we used cytarabine 50 nM and 500 nM in our MSC studies; these concentration were selected because they represent the cytarabine levels reached during low-dose cytarabine (50 nM) and during conventional induction chemotherapy when cytarabine is used in combination with an anthracycline (500 nM) (4). Furthermore, we also tested the effects of the same cytarabine doses on AML cell proliferation in a ^{3}H-thymidine incorporation assay, and cytarabine 50 nM then caused a median reduction of 80 % in the AML cell proliferation and the reduction was more than 50 % for all patients. Whereas 500 nM cytarabine resulted in detectable proliferation for only four patients, and for only one of these patients proliferation was not decreased by more than 95 %.

References

1. Brenner AK, Reikvam H, Bruserud Ø. A Subset of Patients with Acute Myeloid Leukemia Has Leukemia Cells Characterized by Chemokine Responsiveness and Altered Expression of Transcriptional as well as Angiogenic Regulators. *Frontiers in immunology* (2016) **7**: 205.

2. Bruserud Ø, Reikvam H, Fredly H, Skavland J, Hagen KM, van Hoang TT, et al. Expression of the potential therapeutic target CXXC5 in primary acute myeloid leukemia cells - high expression is associated with adverse prognosis as well as altered intracellular signaling and transcriptional regulation. *Oncotarget* (2015) **6**: 2794-811.
3. Ryningen A, Ersvær E, Øyan AM, Kalland KH, Vintermyr OK, Gjertsen BT, et al. Stress-induced in vitro apoptosis of native human acute myelogenous leukemia (AML) cells shows a wide variation between patients and is associated with low BCL-2:Bax ratio and low levels of heat shock protein 70 and 90. *Leuk Res* (2006) **30**: 1531-40.
4. Hubeek I, Kaspers G, Ossenkoppele G, Peters G. Deoxynucleoside analogs in cancer therapy. *Cancer Drug Discovery and Development* (2006): 119-52.
